# Publisher Correction: Multicolor multiscale brain imaging with chromatic multiphoton serial microscopy

**DOI:** 10.1038/s41467-019-10225-w

**Published:** 2019-05-09

**Authors:** Lamiae Abdeladim, Katherine S. Matho, Solène Clavreul, Pierre Mahou, Jean-Marc Sintes, Xavier Solinas, Ignacio Arganda-Carreras, Stephen G. Turney, Jeff W. Lichtman, Anatole Chessel, Alexis-Pierre Bemelmans, Karine Loulier, Willy Supatto, Jean Livet, Emmanuel Beaurepaire

**Affiliations:** 1 Laboratory for Optics and Biosciences, Ecole polytechnique, CNRS, INSERM, IP Paris, Palaiseau, 91128 France; 2 Sorbonne Université, INSERM, CNRS, Institut de la Vision, 17 rue Moreau, Paris, 75012 France; 3 Cold Spring Harbor Laboratory, Cold Spring Harbor, 11724 NY USA; 4 Department of Computer Science and Artificial Intelligence, University of the Basque Country, San Sebastian, 20018 Spain; 5 IKERBASQUE, Basque Foundation for Science, Bilbao, 48013 Spain; 6 Donostia International Physics Center (DIPC), San Sebastian, 20018 Spain; 7 Center for Brain Science and Department of Molecular and Cellular Biology, Harvard University, Cambridge, 02138 MA USA; 8 Neurodegenerative Diseases Laboratory, Molecular Imaging Research Center, Institut de Biologie François Jacob, CEA, CNRS, Université Paris-Sud, Fontenay-aux-Roses, 92265 France

**Keywords:** Microscopy, Neural circuits, Multiphoton microscopy

Correction to: *Nature Communications* 10.1038/s41467-019-09552-9, published online 10 April 2019.

The original version of this Article contained errors in the author affiliations.

Affiliation 4 incorrectly read ‘University of the Basque Country (Ikerbasque), University of the Basque Country and Donostia International Physics Center, San Sebastian 20018, Spain.’

Also, the affiliations of Ignacio Arganda-Carreras with ‘IKERBASQUE, Basque Foundation for Science, Bilbao, 48013, Spain’ and ‘Donostia International Physics Center (DIPC), San Sebastian, 20018, Spain’ were inadvertently omitted.

Additionally, the third sentence of the first paragraph of the Results section entitled ‘Multicontrast organ-scale imaging with ChroMS microscopy’ incorrectly read ‘For example, one can choose λ1 = 850 and λ2 = 110 nm for optimal two-photon excitation of blue and red chromophores.’ The correct version reads ‘λ2 = 1100 nm’ instead of ‘λ2 = 110 nm’. These errors have now been corrected in the PDF and HTML versions of the Article.

