# Supplementary material for: Multicolor multiscale brain imaging with chromatic multiphoton serial microscopy
Source: Nat Commun. 2019 Apr 10;10:1662. doi: 10.1038/s41467-019-09552-9 (PMC6458155; doi:10.1038/s41467-019-09552-9)

Supplementary information for:

Multicolor multiscale brain imaging with chromatic multiphoton serial microscopy

Lamiae Abdeladim et al

Supplementary Figure 1 | High-resolution multicolor anatomical views acquired with ChroMS microscopy

Coronal multicolor anatomical maps in the olfactory bulb (a), forebrain (b), caudal forebrain and brainstem (c), and cerebellum (d). MOB: main olfactory bulb, AOB: accessory olfactory bulb, gl: glomerular layer, opl: outer plexiform layer, CTX: cortex, CC: corpus callosum, CP: caudate putamen, DG : dentate gyrus, CBN: cerebellar nuclei, CBX: cerebellar cortex, gr: granular layer, mol: molecular layer, arb: arbor vitae. Scale bars: 200μm.

Supplementary Figure 1 | High-resolution multicolor anatomical views acquired with ChroMS microscopy

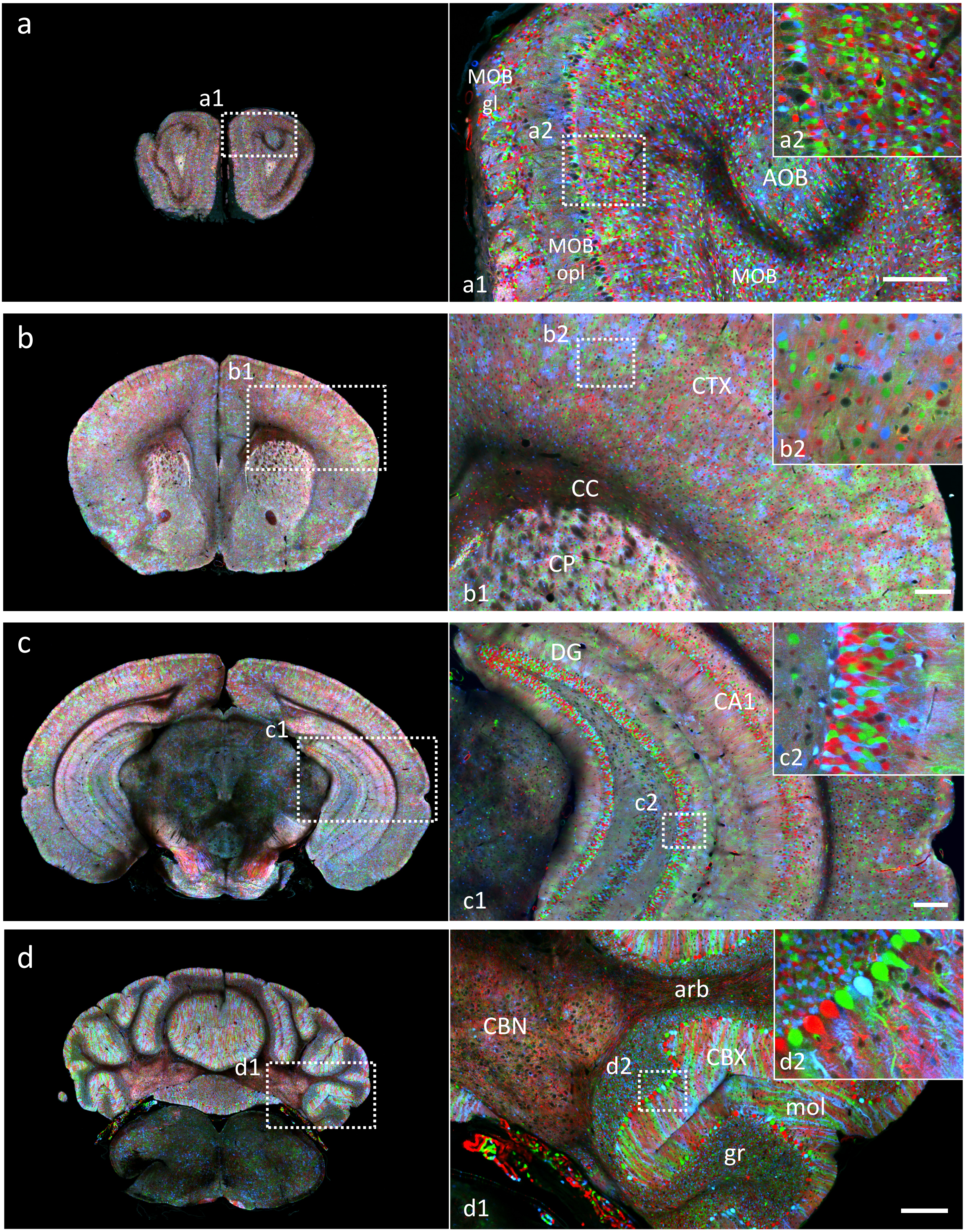

Supplementary Figure 2 | Pre-processing steps for combinatorial multicolor data

(a) Ternary color diagrams before (left) and after (right) color processing. Color processing consists of applying a linear transform to uniformly stretch the [*R*max *G*max *B*max] triangle in the color space so that Bmax is projected to the ‘pure blue’ (0,0,1) position while maintaining the RGB color ratio. (b-c) Brainbow labeled cortical glial cells before (b) and after (c) color processing. Scale bar: 100 μm. (d-e) Mosaics from Brainbow labeled cortical tissue before (d) and after (e) combined flat-field correction and color processing. Scale bar: 300 μm.

Supplementary Figure 2 | Pre-processing steps for combinatorial multicolor data

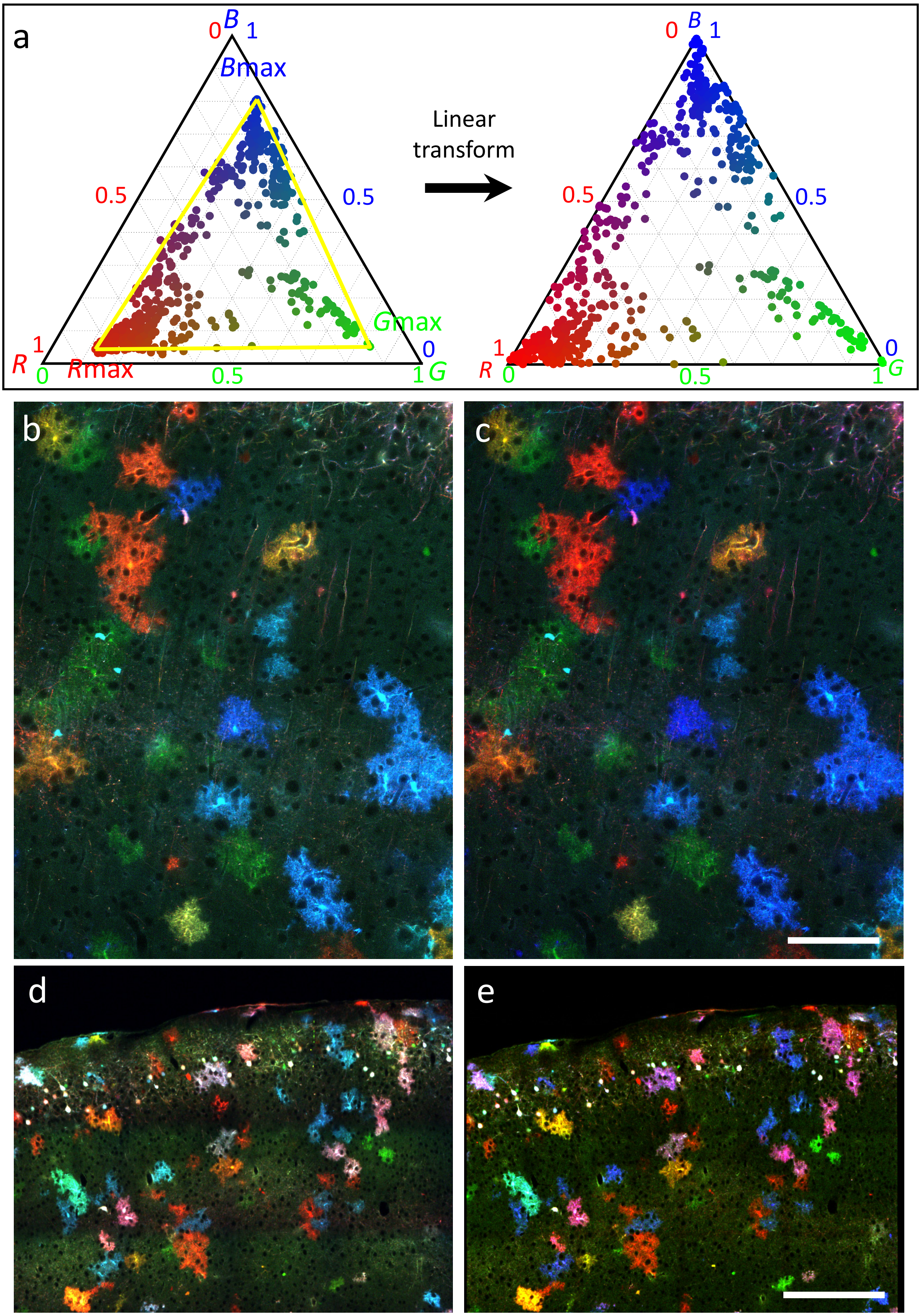

Supplementary Figure 3 | Examples of detected astrocyte color combinations

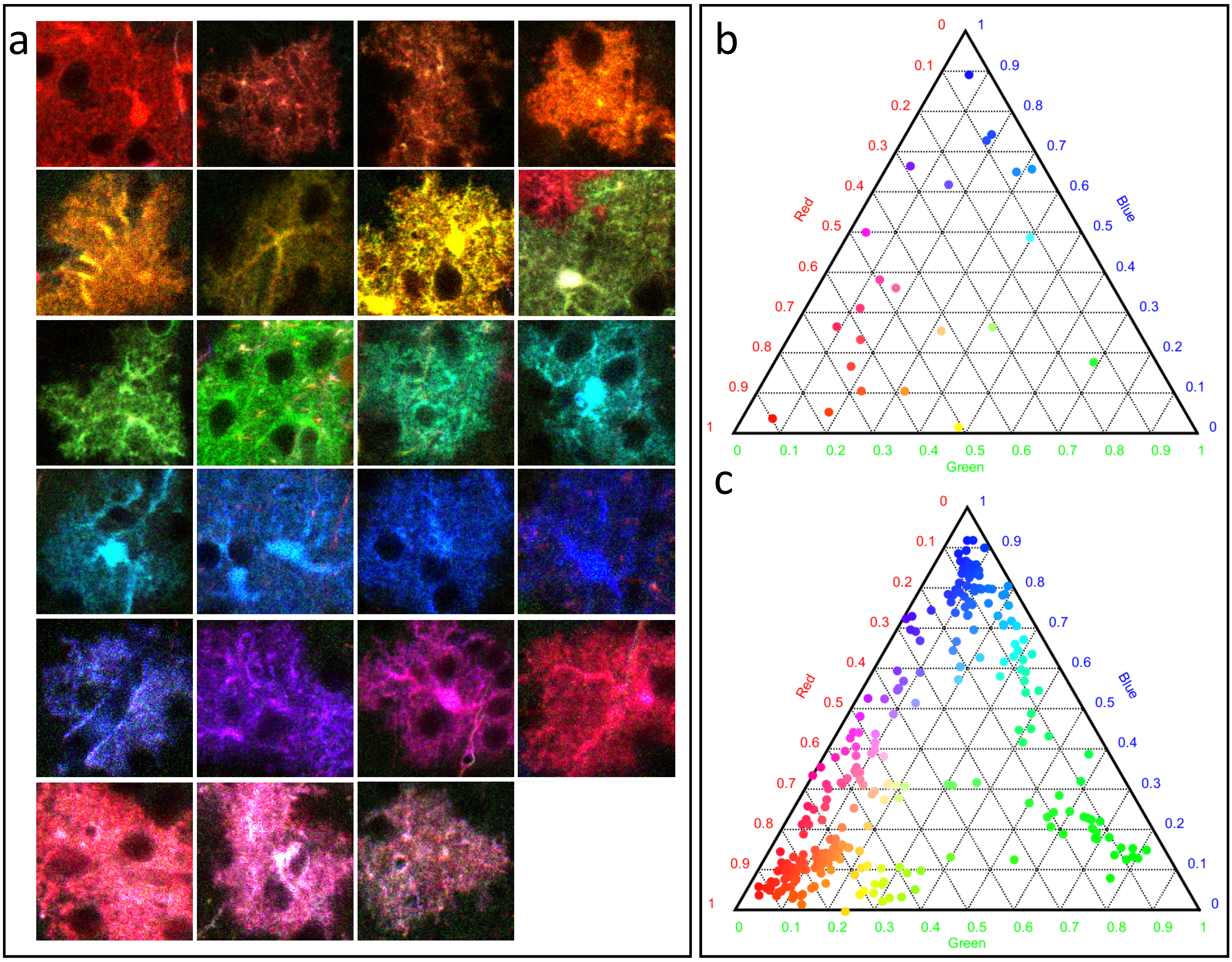

(a) Examples of 23 astrocytes labeled with distinct color combinations detected in the 1.2×2×2 mm^3 volume presented in Fig 3. Shown are crops from the original dataset preprocessed as explained in Suppl Fig S2. (b) Positions of the 23 astrocytes in a ternary color plot. (c) Colorimetric positions of the 261 astrocytes clusters detected in the imaged volume.

Supplementary Figure 4 | Anatomical annotations and cell classification in high-resolution 3D volumes

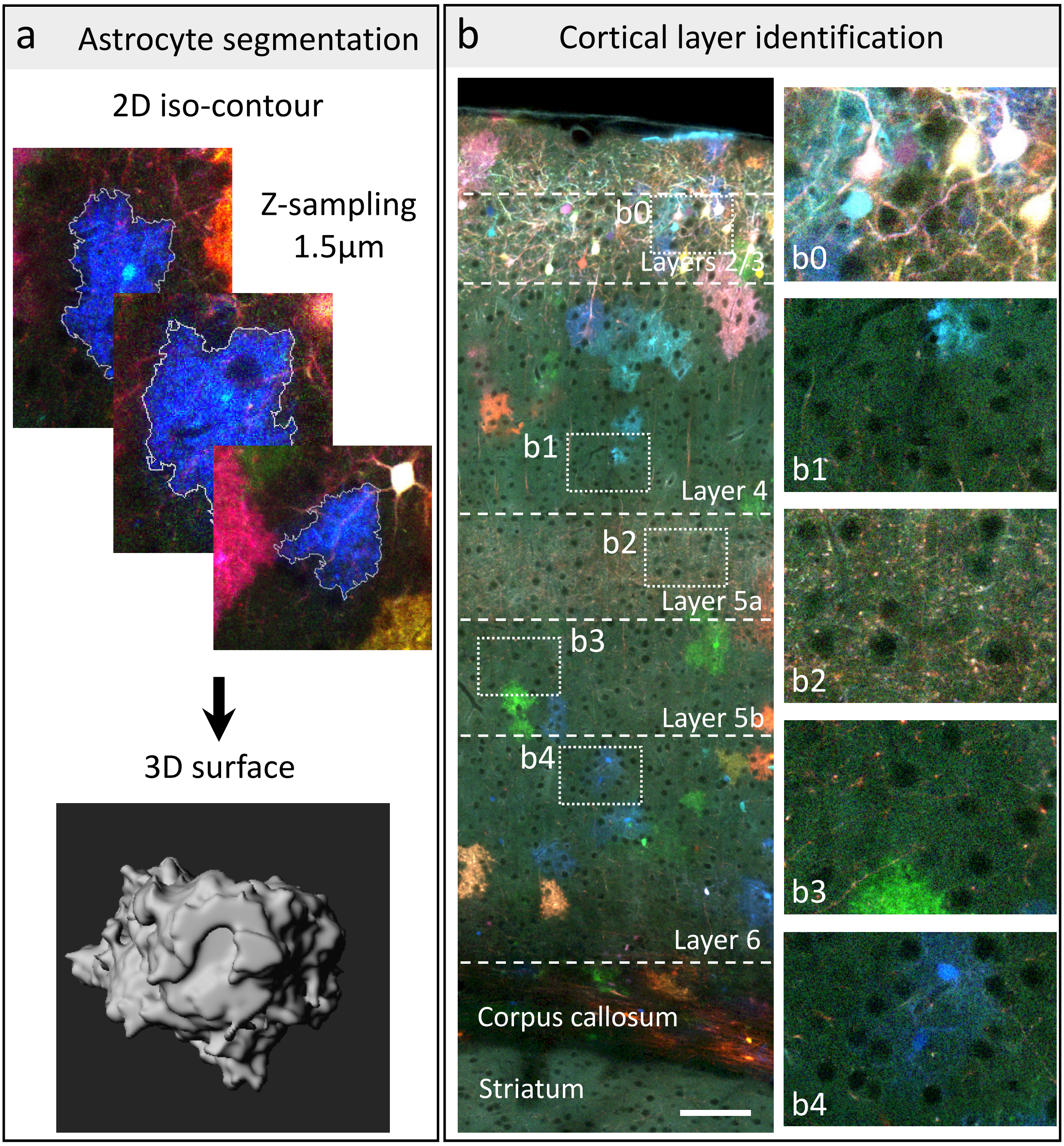

A color-cluster is defined as an ensemble of astrocytes expressing the same color combination and continuously in contact with each other. Somata included within astrocytic domains as well as those located at the astrocytes' periphery were considered for the astroglial network reconstruction. (a) Individual astrocyte segmentation. 2D cell contours are traced in each plane of the corresponding z-stack and a 3D surface is generated by interpolating all the successive 2D contours. (b) Criteria used for cortical layer assignment of labeled astrocytes. Layers 2/3 are identified by the presence of neurons labeled by in utero electroporation at E15 (b0). Layer 5a is identified by the presence of visible projections from upper layers pyramidal neurons (b2). Layer 4 astrocytes included in cortical layer analysis were located in the lower layer 4 region presenting small and densely packed cell bodies (b1). Layers 5b and 6 were discriminated based on cell size and density (b3-b4) and distance to the corpus callosum. Scale bar: 100 µm.

Supplementary Figure 5 | Astrocyte-astrocyte contact processing workflow

(a) Interface segmentation from a color-segregated astrocyte pair. (b) Representation of the astrocyte pair interface in the Voronoi frame. In grey: Voronoi plane. In black: interface plane. Binary color bar represents the bias parameter (ratio of interface points above the Voronoi plane). (c) 3D Surface fitting in the interface frame. Sampling: 1 µm x 1 µm. (d) Projection of interface points onto the interface plane (left). Contour points are defined as the most distant points on each interface z-stack plane (right). (e) 3D interface reconstruction and derived height and gradient maps. 3x3 median filtering is applied on the 1 µm x 1 µm surface fit to generate the height map. Black lines on the height map represent altitude isolines referenced to the interface plane. Gradient map is computed as $(\partial w/\partial u).(\partial w/\partial u) + (\partial w/\partial v).(\partial w/\partial v)$. Axis units : µm

Supplementary Figure 5 | Astrocyte-astrocyte contact processing workflow

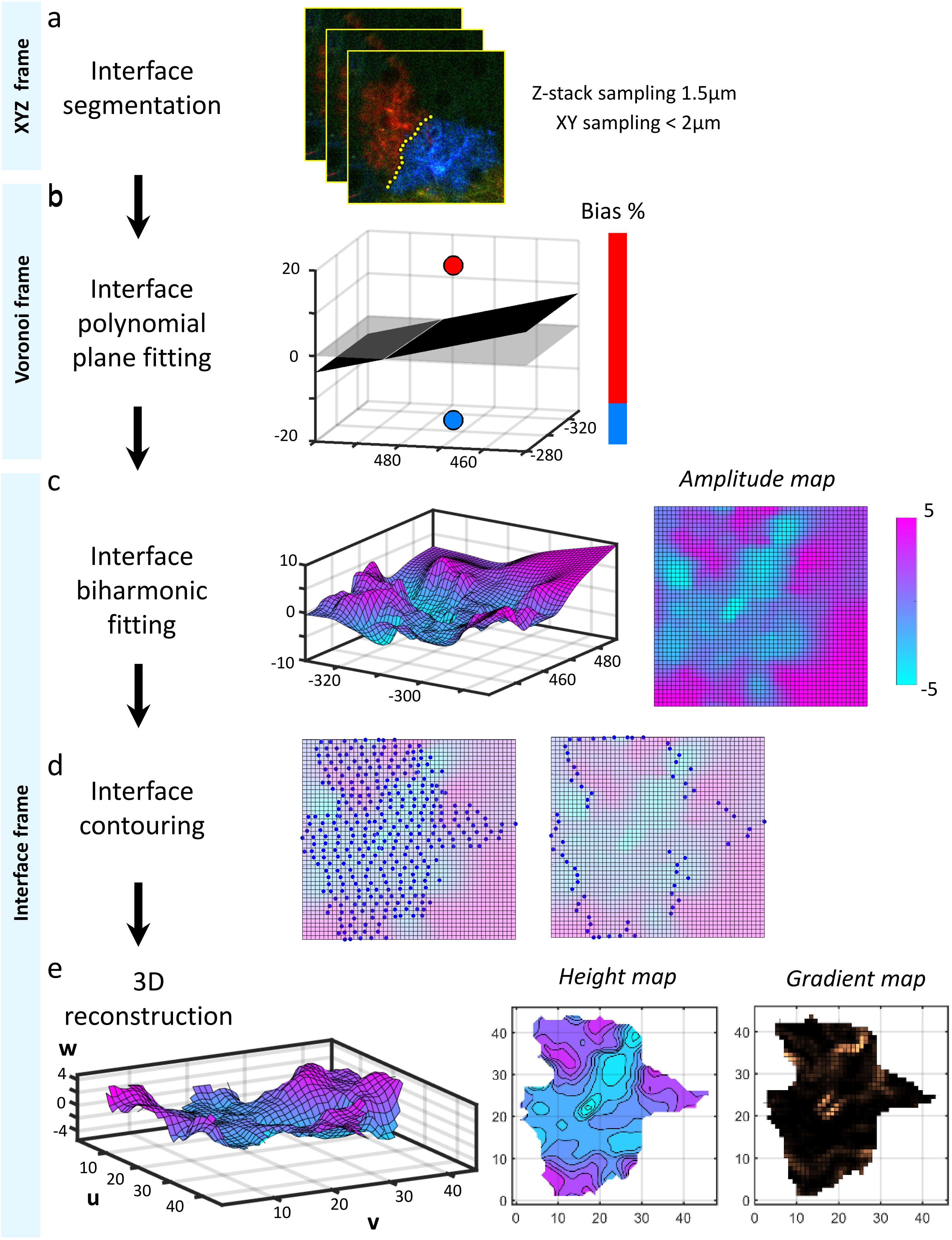

Supplementary Figure 6 | Astrocyte-astrocyte contact analysis

Inventory of analyzed astrocyte color pairs. *Orientation*: angle between the Voronoi plane and the interface plane in the Voronoi frame. *Bias*: Ratio of interface points above the Voronoi plane. *Distance*: distance between the two astrocyte somata. *Surface*: interface area. *MSD*: mean square distance parameter computed as the total sum of square amplitudes divided by the interface area. *Wiggliness*: total sum of the gradient parameter (as defined in Suppl Fig 6) divided by the restricted interface area. Restricted interface area is defined as the interface area with a finite gradient parameter value, i.e borders of the interface area are excluded.

Supplementary Figure 6 | Astrocyte-astrocyte contact analysis

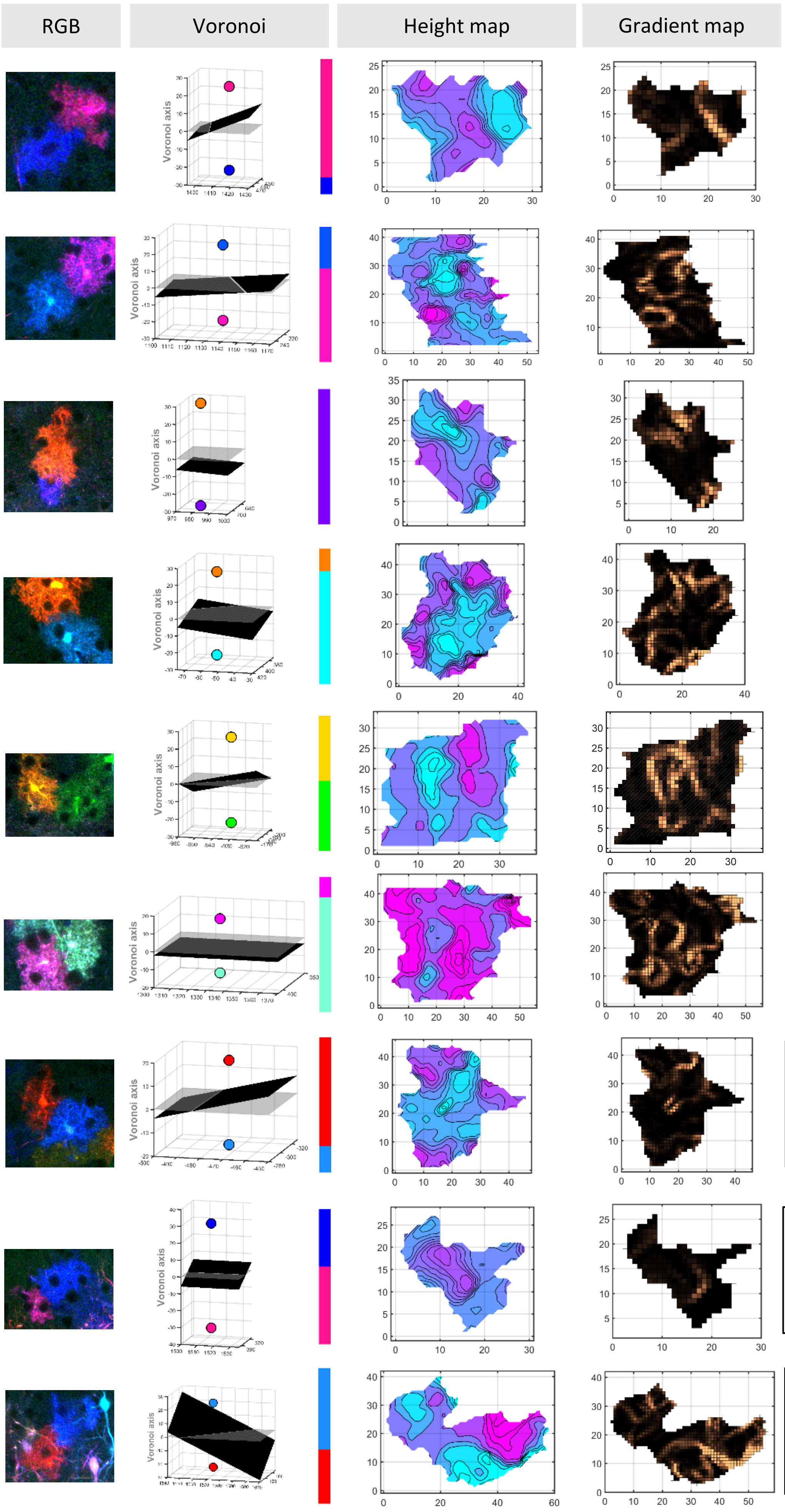

Parameters

Orientation : 22.7° / Bias : 12%
Distance : 47 µm
Surface : 443 µm²
MSD : 4.1 / Wiggliness : 0.64

Orientation : 6.2° /Bias : 31%
Distance : 44 µm
Surface : 1282 µm²
MSD : 3.9 / Wiggliness : 0.32

Orientation : 3.7° / Bias : 0%
Distance : 59 µm
Surface : 504 µm²
MSD : 3.3 / Wiggliness : 0.47

Orientation : 11.1° / Bias : 16%
Distance : 50 µm
Surface : 1123 µm²
MSD : 5.8 / Wiggliness : 0.51

Orientation : 20.8° / Bias : 48%
Distance : 49 µm
Surface : 897 µm²
MSD : 5.41 / Wiggliness : 0.80

Orientation : 1° / Bias : 15%
Distance : 30 µm
Surface : 1603 µm²
MSD : 25.1 / Wiggliness : 0.69

Orientation : 13.3° / Bias : 19%
Distance : 36 µm
Surface : 1158 µm²
MSD : 4.2 / Wiggliness : 0.23

Orientation : 19.5° / Bias : 43%
Distance : 62 µm
Surface : 379 µm²
MSD : 1 / Wiggliness : 0.18

Orientation : 33.8° /Bias : 39%
Distance : 48µm
Surface : 1196 µm²
MSD : 16 / Wiggliness : 1.4

Supplementary Figure 7 | Dissection of neural projections with ChroMS microscopy

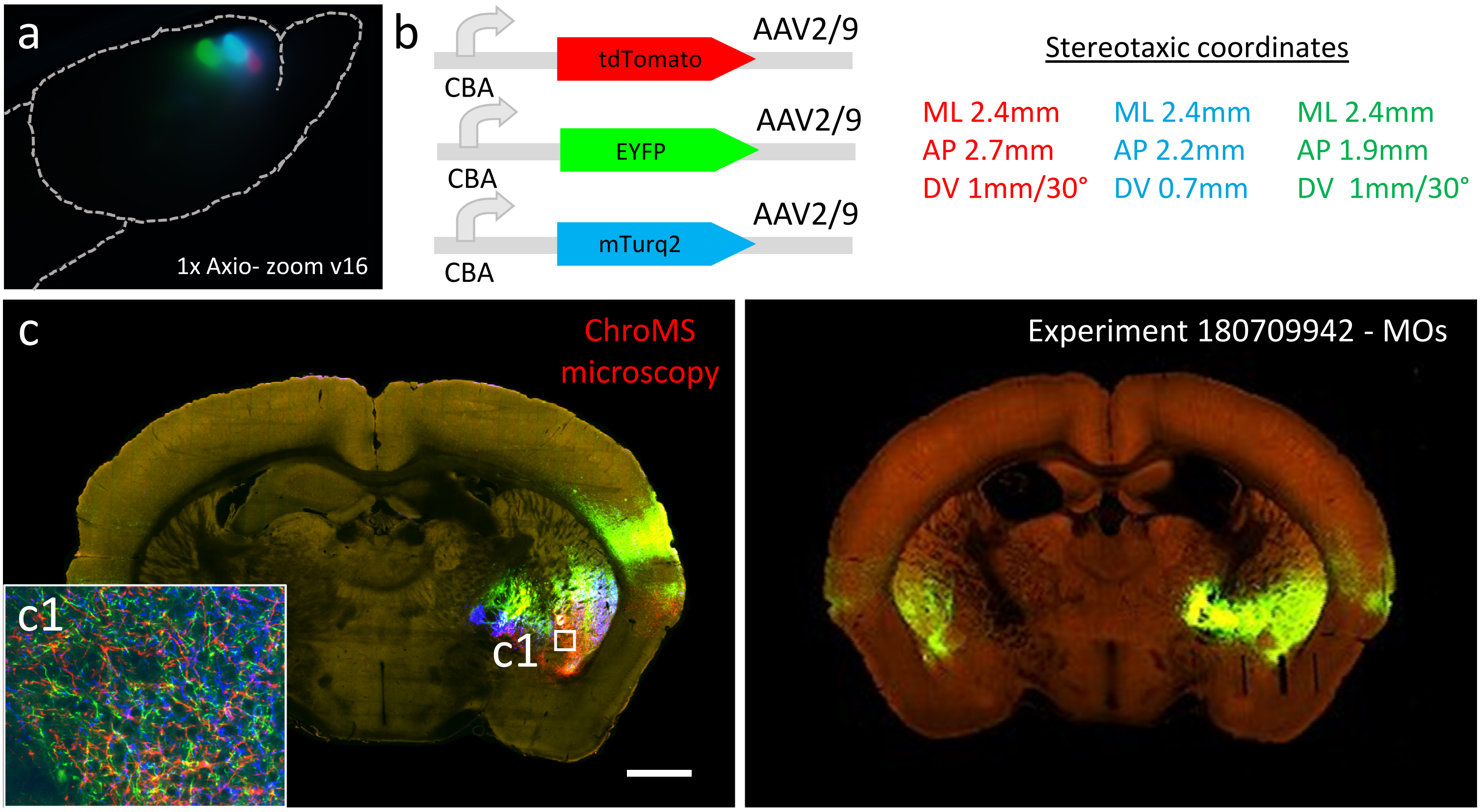

(a) Epifluorescence whole-brain snapshot of tricolor injection areas. Image acquired on a commercial Zeiss Axio-zoom microscope. (b) Viral constructs and injection coordinates used in tricolor multiplex AAV labeling (c) Multicolor 2D coronal section acquired with ChroMS microscopy (left) and equivalent monochrome 2D coronal section (right) from the Allen Connectivity database [http://connectivity.brain-map.org/] shows the capability of the ChroMS strategy to resolve axonal projections originating from areas injected with distinct viral tracers and to visualize their fine interdigitation in target areas. Scale bar: 1 mm.

Supplementary Figure 8 | High-resolution segmentation of fluorescence signal

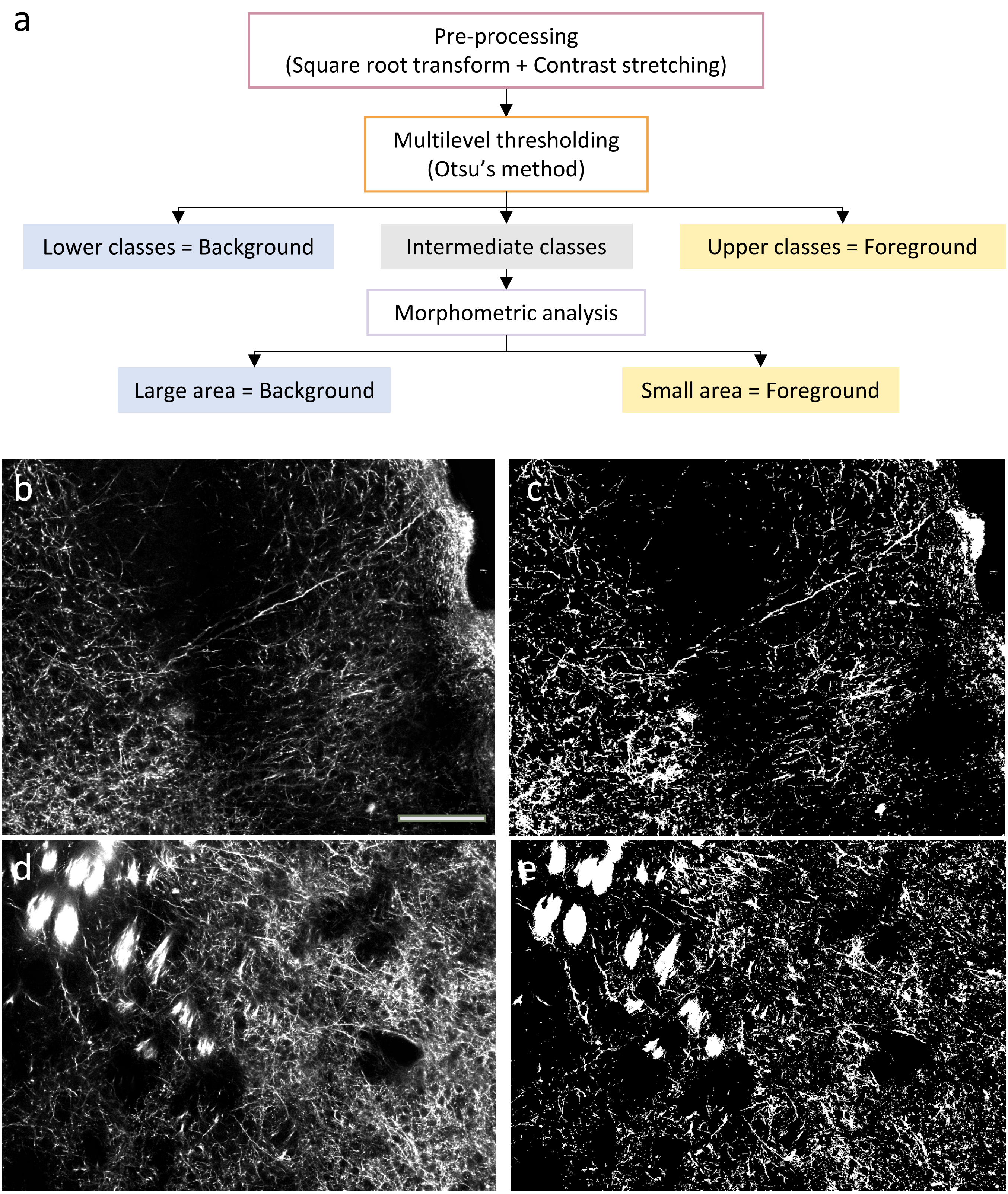

(a) Layout of the segmentation algorithm used to process tractography data (Fig 6). Fluorescence is separated from background using intensity-based clustering combined with morphometric analysis. (b-e) Single-channel images from the multiplexed connectivity dataset (left) and corresponding fluorescence binary masks (right). Scale bar: 200 µm.

Supplementary Figure 9 | Quantitative topographical analysis workflow

Summary of processing steps for multiplexed analysis of axonal projections. Pre-processing steps consist of flat-field correction of individual tiles followed by stitching. Spectral unmixing is performed by applying a linear transform in the color space with barycentric initial conditions (see Methods). The segmentation algorithm (presented in Suppl Fig 8) generates high-resolution fluorescence binary masks. Positive pixels from each color-exclusive binary mask are summed over 24 μm x 24 μm (i.e. 30x30 pixels) to compute projection strength maps. Projection maps are then merged into quantitative interdigitation maps. Color maps displayed in regular contrast (top) allow for direct visualization of interdigitation from strong projections. Heptachromic contrast (bottom) highlights interdigitation from weak axonal projections.

Supplementary Figure 9 | Quantitative topography analysis workflow

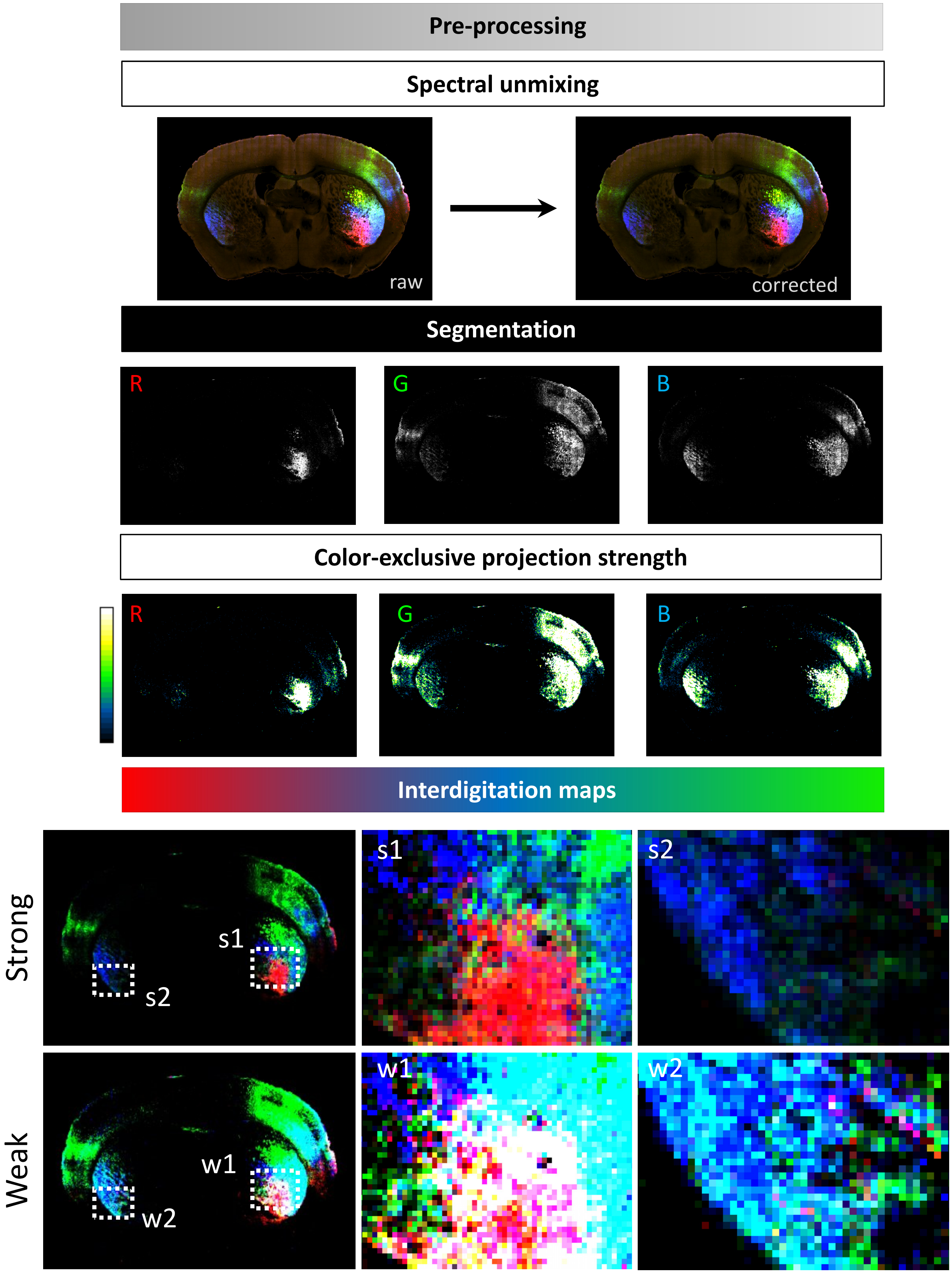

Supplementary Figure 10 | Exclusive projection confidence maps

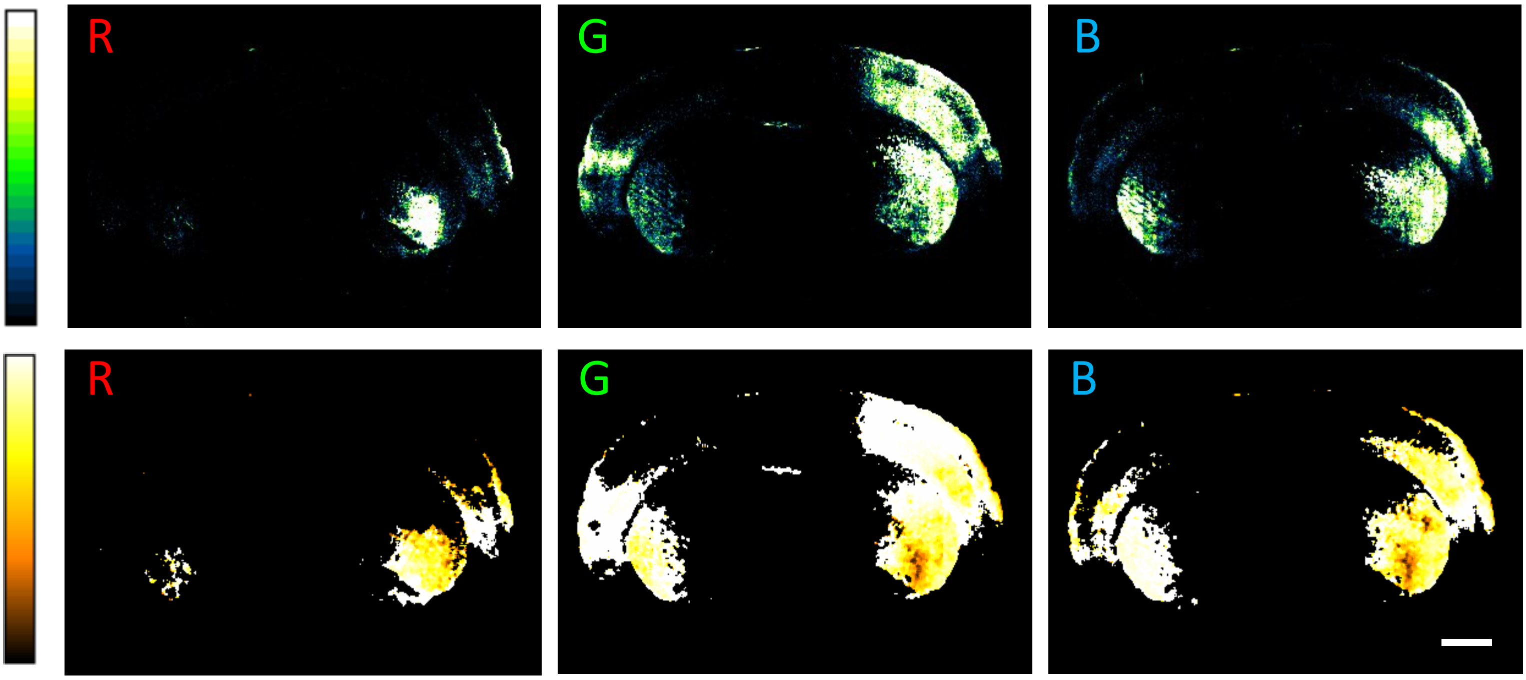

Top: Red, green, and blue exclusive projection strength maps. Signal intensity is represented in arbitrary units from weak (dark blue) to strong (white) projections. Bottom: Red, green and blue exclusive projection confidence maps. Exclusive projection confidence maps are computed as the ratio between projection strength maps calculated with exclusive HR masks over projection strength map calculated with raw HR masks. Exclusive projection confidence maps therefore account for potential errors due to excluding dual-color pixels from interdigitation analysis. Signal intensity scales range from 0% (dark) to 80% (light). Pixel size: 24 µm x 24 µm. Scale bar: 1mm.

Supplementary Figure 11 | Analysis of projection terminals in the caudoputamen

(a) Interdigitation maps computed across the caudoputamen and corresponding interdigitation diagrams. Interdigitation diagrams quantify interdigitation relative proportions following super-pixel classification into a single (red, green, blue), dual (yellow, cyan, magenta) or triple projection category. (b-c) Graphs displaying interdigitation relative proportions across the caudoputamen. (d) Interdigitation results are robust to changes of super-pixel size across a range of relevant sizes (20 to 40 μm). See also Supplementary Movie 14.

Supplementary Figure 11 | Analysis of projection terminals in the caudoputamen

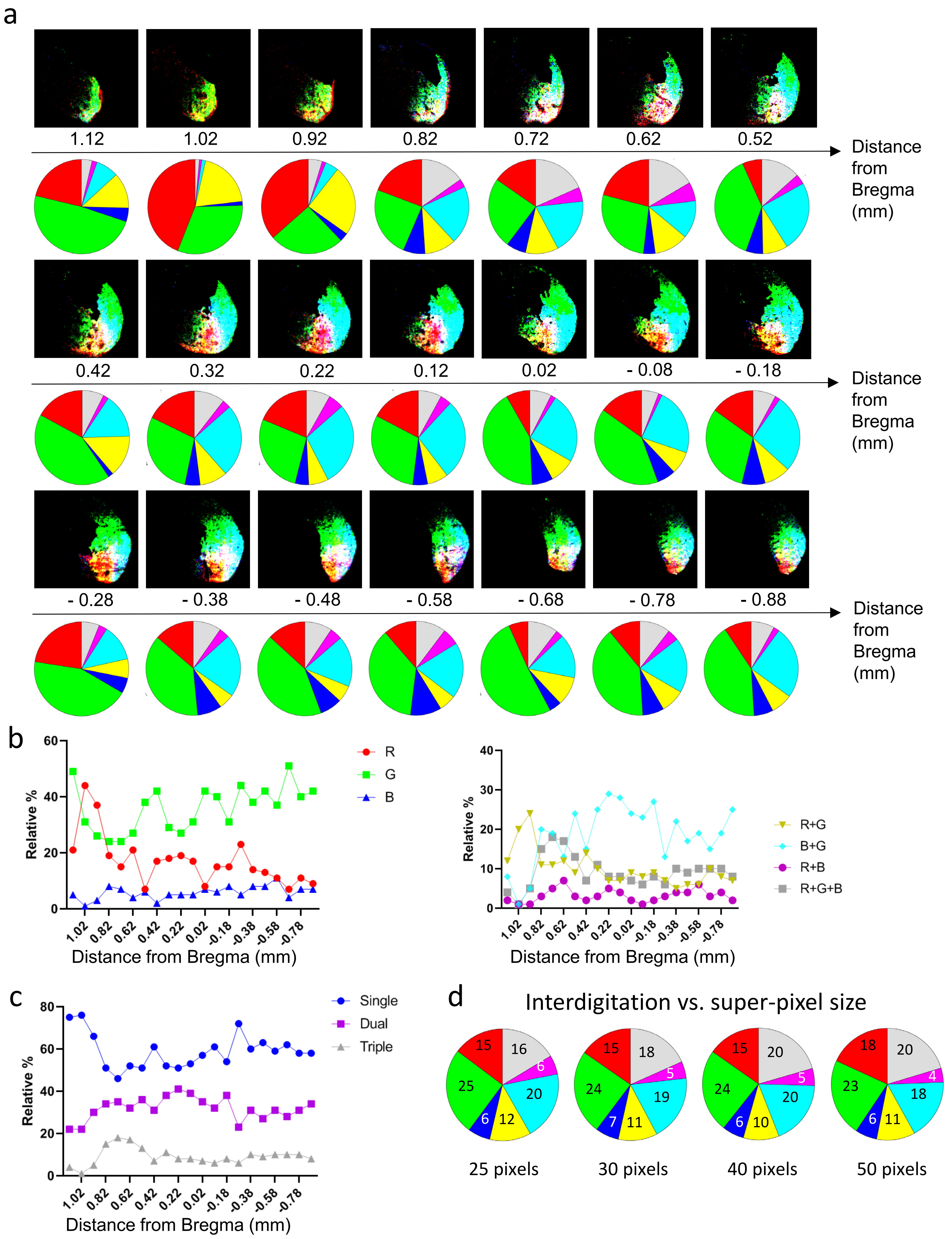

Supplementary Figure 12 | Analysis of corticofugal fiber interdigitation in the striatum

(a) Interdigitation maps and corresponding interdigitation diagrams computed across the striatum on two fiber tracts labelled with distinct colors. The caudoputamen was manually segmented within the striatal region to remove passing fiber tracts and keep mostly axonal arbors. Interdigitation diagrams quantify interdigitation relative proportions following super-pixel classification into a single (green, blue) or dual (cyan) projection category. (b) Graphs displaying fiber tract interdigitation throughout the striatum and showing tract segregation in the anterior part, followed by fiber interdigitation when entering the external pallidus segment. (c) Dual-color images showing segregated fibers +0.42 mm from Bregma (left) and interdigitated fibers at -0.68 mm from Bregma (right). Note: residual post-unmixing spectral bleedthrough systematically located at the center of fiber tracts due to saturating signals have been manually removed. Scale bars: 100 µm. See also Supplementary Movie 14.

Supplementary Figure 12 | Analysis of corticofugal fiber interdigitation in the striatum

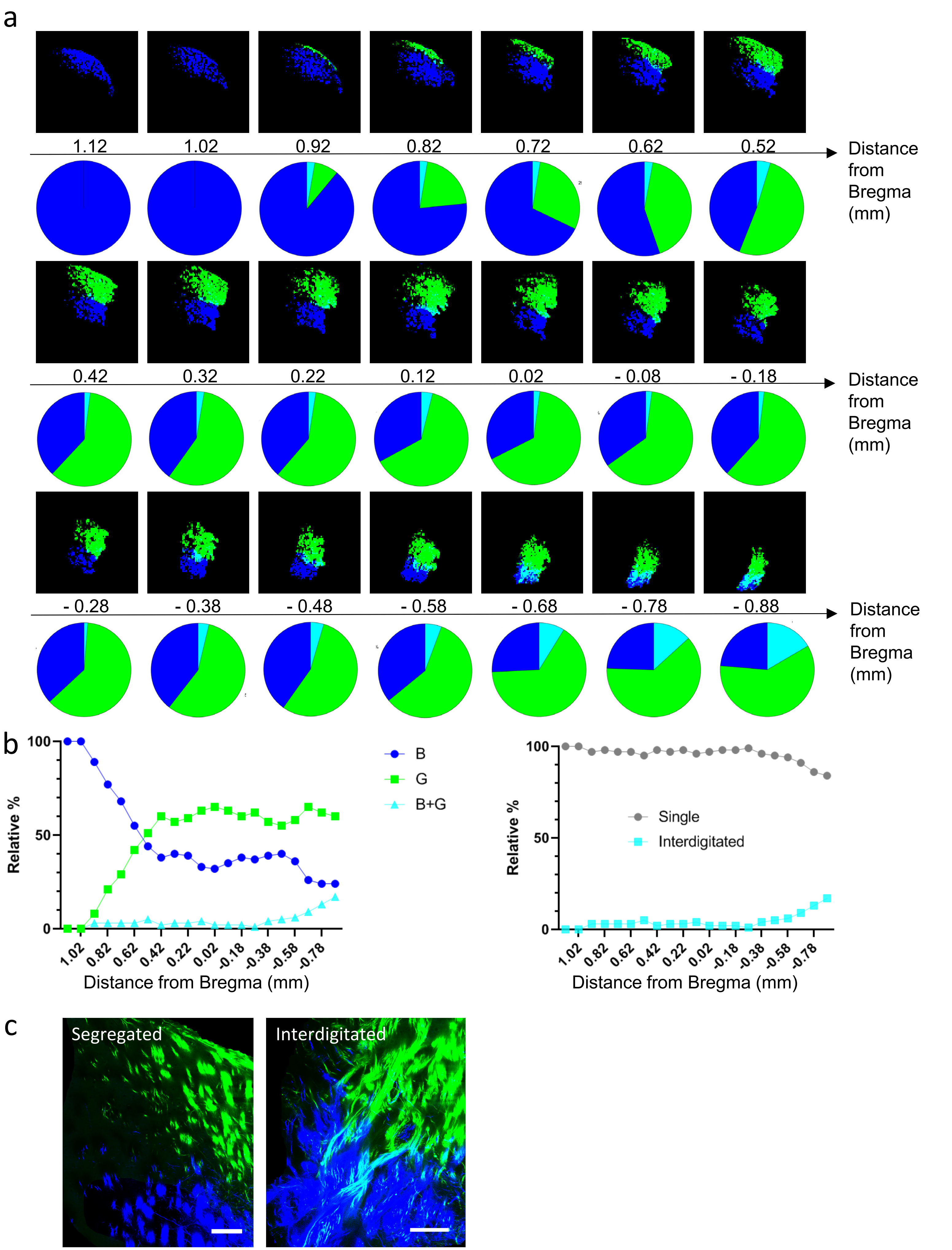

Supplementary Figure 13 | ChroMS setup: sample holder and excitation wavelengths used for multimodal imaging

a

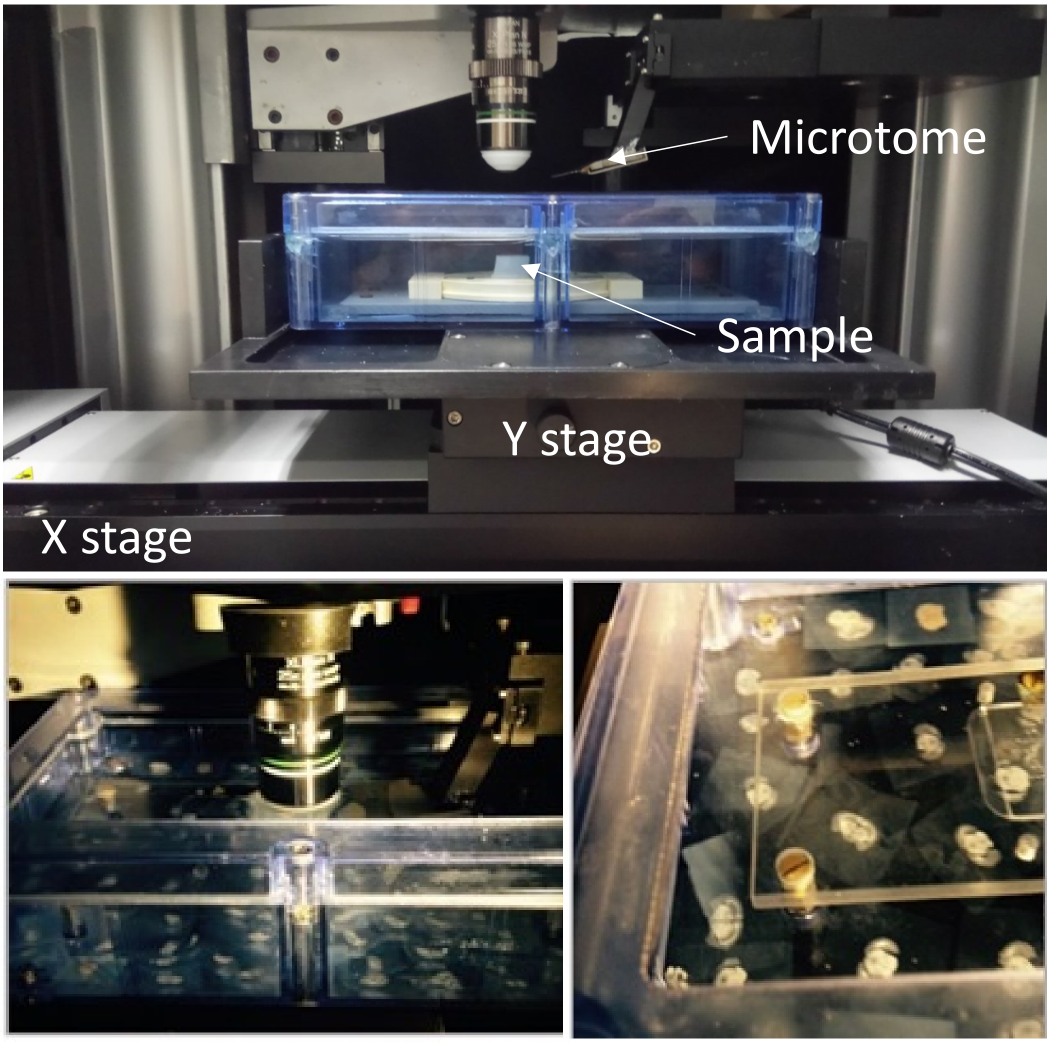

b

	Detection channels			
Excitation wavelengths	UV	Blue	Yellow/Green	Red/IR
**850 nm/ 1100 nm**	THG/366 nm	Fluo/SHG	Fluo/SHG	Fluo
**832 nm/ 1090 nm**	THG/363 nm	Fluo/SHG	Fluo/SHG	CARS/676 nm

(a) Pictures of the ChroMS microscopy setup sample stage. Obj: objective. (b) Accessible wavelength combinations. In bold, combinations demonstrated in the present paper. In grey, other accessible combinations. The 850 nm/1100 nm and 832 nm/ 1090 nm combinations both enable simultaneous harmonic and fluorescence imaging. Fluo: fluorescence, SHG: second harmonic generation, THG: third harmonic generation.

Supplementary Figure 14 | Optimization of the scanning system

(a-c) Ray-tracing Zemax simulations used for the design and optimization of the scanning system in the ChroMS setup. We found that B coated optics (Thorlabs, a) displayed higher performances than their C coated counterparts (b) in terms of lateral chromatic aberration and field curvature. Besides, we obtained higher performances with a Plössl-lens design for the scan lens (c). (d) Lateral chromatic aberration map measured after the objective with KTP nanoparticles at 850 and 1100 nm. (e) Calculated theoretical wavelength-mixing efficiency across the field of view based on the chromatic aberration map. (f) Measured intensity profile for the green channel. The intensity profile was obtained by averaging all green channel images from an acquired multicolor dataset and then applying a large kernel Gaussian blur. The measured profile takes into account both the efficiency of the wavelength-mixing process across the field of view and any eventual inhomogeneity of the detection. The measured profile is used as a calibration to flat-field correct all individual tiles.

Supplementary Figure 14 | Optimization of the scanning system

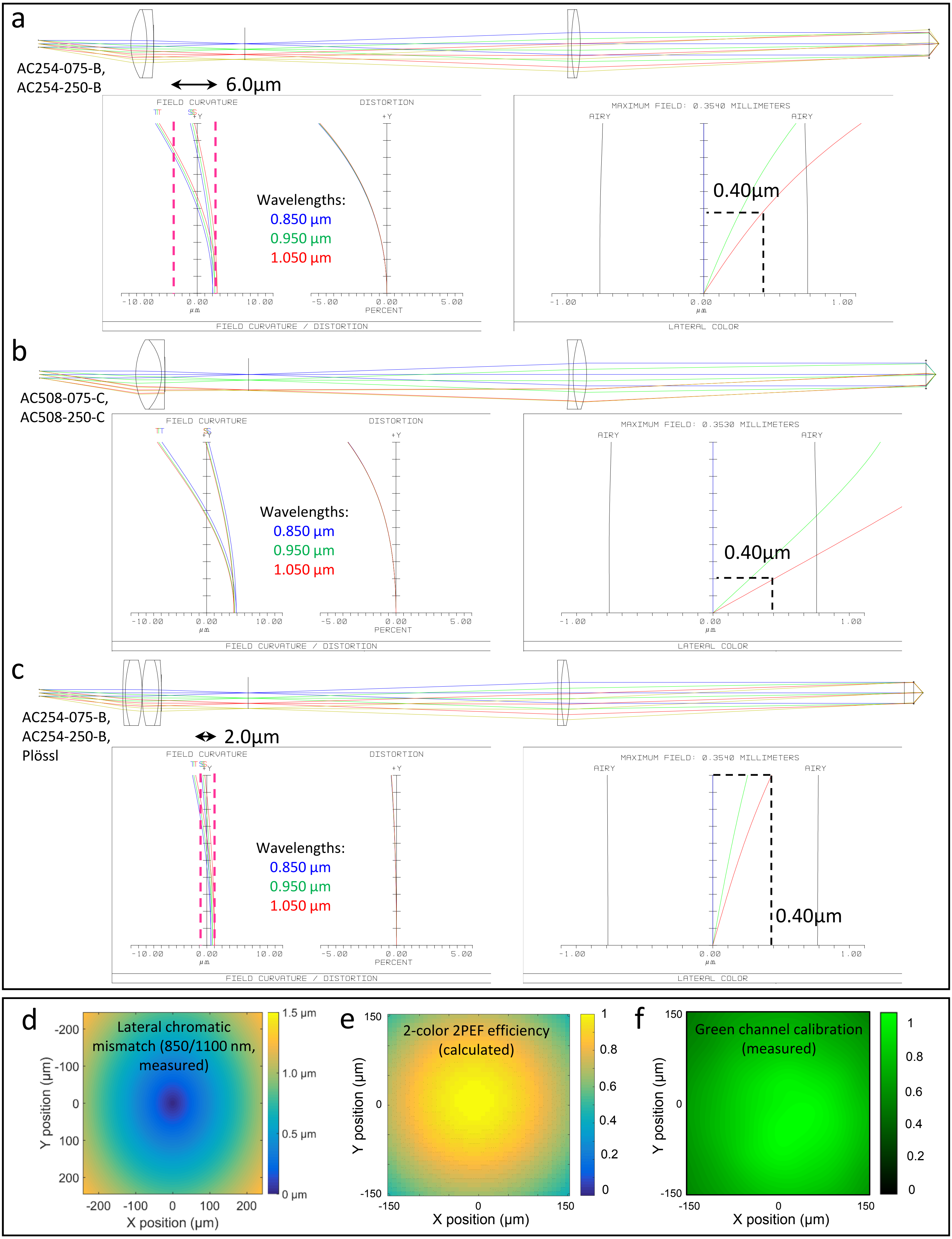

Supplement: Supplementary file 1 — Supplementary Information [file 41467_2019_9552_MOESM1_ESM.pdf]
