# Supplementary material for: Multicolor multiscale brain imaging with chromatic multiphoton serial microscopy
Source: Nat Commun. 2019 Apr 10;10:1662. doi: 10.1038/s41467-019-09552-9 (PMC6458155; doi:10.1038/s41467-019-09552-9)
Supplement: Supplementary file 3 — Description of Additional Supplementary Files [file 41467_2019_9552_MOESM3_ESM.pdf]

Description of Additional Supplementary Files

File Name: Supplementary Movie 1
Description: Principles of ChroMS microscopy. Principles of the wavelength-mixing trichromatic acquisition scheme combined with serial sectioning to achieve whole-brain multicolor imaging.

File Name: Supplementary Movie 2
Description: Whole-brain multicolor imaging with ChroMS microscopy 3D rendering of an entire Brainbow (*CAG-Cytbow;Nestin-Cre)* mouse brain (presented in Fig. 1) acquired with ChroMS microscopy. ChroMS 2D images were downsampled by a factor of 10.

File Name: Supplementary Movie 3
Description: Whole Brainbow mouse brain acquired with ChroMS microscopy. Movie showing the 92 consecutive coronal sections acquired for the 3D reconstruction of the Brainbow (*CAG-Cytbow;Nestin-Cre)* mouse brain presented in Fig. 1. ChroMS 2D images were downsampled by a factor of 10.

File Name: Supplementary Movie 4
Description: High-resolution multicolor anatomical map in the olfactory bulb. 2D ChroMS microscopy optical section acquired in the olfactory bulb with 0.55 µm pixel size, from the Brainbow mouse brain dataset.

File Name: Supplementary Movie 5
Description: High-resolution multicolor anatomical map in the cerebellum. 2D ChroMS microscopy section in the cerebellum with 0.55 µm pixel size, from the *Cytbow* mouse brain dataset.

File Name: Supplementary Movie 6
Description: Multicolor 3D continuous imaging with preserved contrast and resolution over arbitrary depth. Multicolor ChroMS 3D imaging of the 1.2x2x2 mm3 volume of mouse cerebral cortex labeled with *MAGIC Markers* shown in Fig. 3 with 0.4 µm×0.4 µm×1.5 µm voxel size. Resolution, contrast and channel-registration are preserved through the entire dataset.

File Name: Supplementary Movie 7
Description: Large cortical volume (>5mm$^3$) with thousands of color-labeled astrocytes. Fly-through movie across a >5mm3 chromatically-corrected multicolor volume of mouse cerebral cortex labeled with MAGIC Markers and containing thousands color-labeled astrocytes, imaged at 0.4 µm × 0.4 µm ×1.5 µm voxel size.

File Name: Supplementary Movie 8
Description: 3D multicolor imaging of astrocyte arboritic domains and neuronal processes over arbitrary depth. 3D rendering of glial and neuronal processes with 0.4 µm×0.4 µm×1.5 µm multicolor voxel size precision in the cerebral cortex volume labeled with *MAGIC Markers* shown in Fig. 3.

File Name: Supplementary Movie 9
Description: Color-labeled layer 2/3 pyramidal neurons imaged within a large (>5mm$^3$) volume. 3D rendering of the imaged cortical volume (2.6 mm × 2 mm × 1.1 mm) presented in Fig. 5 containing color-labeled layer 2/3 pyramidal neurons and zooming inside the dataset showing how color-contrasts allows to disambiguate intermingled neurites.

File Name: Supplementary Movie 10
Description: 3D tracing of intermingled cortical neurites in a dense environment. 3D view of a densely-labeled cortical area and corresponding traced neuronal arborizations.

File Name: Supplementary Movie 11
Description: Brain-wide 3D projection topography. 3D rendering of the *triple-AAV* labeled mouse

brain presented in Fig 6, showing multiplexed tract imaging. ChroMS microscopy 2D brain images were downsampled by a factor of 10.

File Name: Supplementary Movie 12
Description: Brain-wide multiplexed axonal tract mapping. Movie showing the 82 consecutive coronal sections acquired for the 3D reconstruction of the *triple-AAV* mouse brain presented in Fig. 6. ChroMS 2D images were downsampled by a factor of 10.

File Name: Supplementary Movie 13
Description: High-resolution multiplexed projection imaging. ChroMS microscopy 2D optical section in the forebrain at 0.8 µm×0.8 µm multicolor pixel precision, from the *triple-AAV* mouse brain dataset presented in Fig. 6, showing interdigitation of axonal projections labeled with distinct colors.

File Name: Supplementary Movie 14
Description: Projection interdigitation analysis in the striatal area. Multi-projection imaging throughout the striatum (from 1.12 mm to -0.88 mm distance to Bregma) and corresponding interdigitation map (24 µm × 24 µm super-pixel size) and interdigitation diagram.
